# Low-Rank and Sparse Matrix Recovery for Hyperspectral Image Reconstruction Using Bayesian Learning

**DOI:** 10.3390/s22010343

**Published:** 2022-01-04

**Authors:** Yanbin Zhang, Long-Ting Huang, Yangqing Li, Kai Zhang, Changchuan Yin

**Affiliations:** 1 Beijing Laboratory of Advanced Information Networks, Beijing Key Laboratory of Network System Architecture and Convergence, Beijing University of Posts and Telecommunications, Beijing 100876, China; liyq@bupt.edu.cn (Y.L.); kaizhang@bupt.edu.cn (K.Z.); ccyin@bupt.edu.cn (C.Y.); 2 China Fire and Rescue Institute, Beijing 102202, China; 3 School of Information Engineering, Wuhan University of Technology, Wuhan 430070, China; huanglt08@whut.edu.cn

**Keywords:** hyperspectral images, hyperspectral remoting sensing, Bayesian learning, compressive sensing, low-rank and joint-sparse

## Abstract

In order to reduce the amount of hyperspectral imaging (HSI) data transmission required through hyperspectral remote sensing (HRS), we propose a structured low-rank and joint-sparse (L&S) data compression and reconstruction method. The proposed method exploits spatial and spectral correlations in HSI data using sparse Bayesian learning and compressive sensing (CS). By utilizing a simultaneously L&S data model, we employ the information of the principal components and Bayesian learning to reconstruct the hyperspectral images. The simulation results demonstrate that the proposed method is superior to LRMR and SS&LR methods in terms of reconstruction accuracy and computational burden under the same signal-to-noise tatio (SNR) and compression ratio.

## 1. Introduction

HSI is a collection of hundreds of images that are usually acquired simultaneously in narrow and adjacent spectral bands by airborne sensors [1] or spaceborne spectrometers [2]. HSI combines the traditional two-dimensional remote sensing imaging technology and the optical imaging technology of spectroscopy [3]. Moreover, HSI has achieved the effect of acquiring images and spectra of objects at the same time, which has set off a revolution in the field of remote sensing. In recent years, with the rapid development of precision agriculture, hyperspectral imaging (HSI) technology in hyperspectral remote sensing [4] has been widely used. In precision agriculture, hyperspectral images can be used to monitor drought and flooding in farmlands, pests and diseases, and crop growth, as well as to predict farmland yield. According to the estimated variability from HSI, precision agriculture can improve resource-use efficiency, productivity, quality, profitability, and sustainability of agricultural production, such as by using less irrigation water, fewer pesticides and fertilizers, etc. HSI is also used in many applications besides precision agriculture, including forestry monitoring, natural resource investigation, vegetation observation, food safety, and geological mapping.

However, the acquisition and processing of HSI require a high-sensitivity detector, ultra-long-distance transmission, great computing power, and a huge data storage capacity in order to deal with such huge amounts of data. The HSI data need to be transmitted to ground stations. In this regard, one of the obstacles that researchers have had to face in the past 20 years is that of finding a way to program hyperspectral satellites to reduce the HSI data. Fortunately, HSI data are low-rank [5] and sparse in the transform domain (discrete cosine transform (DCT) or wavelet transform) [6,7,8]. These kinds of data have the features of being low-rank and sparse, which means that the data are redundant, and a small number of datasets can be used to express the entirety of the data. So, we can use these features to reduce the amount of HSI data to be transmitted. This might be a good way to solve the problem.

Recently, many works have focused on modeling, compressing, and reconstructing HSI data using a structured framework. By dividing HSI into many blocks, Zhang et al. [5] introduced an HSI data reconstruction method based on low-rank matrix recovery (LRMR). In particular, for this kind of signal, many new compressive sensing (CS)-based methods [9,10,11] have been proposed [6,8,12,13,14,15,16,17,18,19]. Golbabaee et al. [6] simultaneously reconstructed HSI data with a low-rank and joint-sparse (L&S) structure by assuming that HSI data are low-rank and using a spatially joint-sparse wavelet representation. Zhang et al. [12] showed that the block sparse Bayesian learning (bSBL) algorithm has good recovery performance for data with a spatial block structure (such as an L&S structure). However, most of the existing research focused on low-rank structure reconstruction methods or HSI data denoising methods, and there are no methods for integrating the process of HSI acquisition and combining a sparse structure reconstruction method to reconstruct HSI data.

In this paper, a method based on the bSBL framework is proposed to compress, transmit, and reconstruct the entirety of HSI data. Here, each sub-matrix of multi-channel data is collected, compressed, and transmitted to the ground processing center through push-broom imaging. The proposed method not only combines the L&S structure priors on HSI data and the appropriate priors on hyperparameters, but also uses the information of the main components of the data. Firstly, we assume that the covariance matrix of the data is a diagonal matrix, and then obtain the initial value of the hyperparameter. Finally, an EM-like method is used to obtain the best reconstruction of the HSI data. The simulation results show that the proposed method has better performance than other existing methods.

We highlight the following main contributions:

(1) The proposed method gives the structure of the covariance matrix of the L&S signals, models HSI data with the L&S structure, and utilizes the CS and Bayesian learning methods to compress and reconstruct HSI data.
(2) In the reconstruction part, the proposed method makes use of the relationship between multiple dimensions of high-dimensional data and combines data reconstruction with HSI data acquisition. It can be used to realize the segmented acquisition, compression, and transmission of HSI data so as to reduce the amount of calculation and data transmission that must be performed in the satellite.
(3) We demonstrate the superior performance of the proposed method in comparison with state-of-the-art alternatives by conducting experiments on both synthetic signals and real signals.

The rest of this paper is arranged as follows: Section 2 introduces two 2D reconstruction methods for HSI data. Section 3 introduces the L&S data compression and reconstruction method combined with the acquisition mode. Section 3.1 introduces a model of the problem, and Section 3.2 proposes the HSI reconstruction method based on push-broom imaging. Section 3.3 introduces the simulation results. Discussion is given in Section 4. Finally, this work is concluded in Section 5.

Notation: p(A)∼N(0,Σ) denotes the probability density function of A, which follows a Gaussian distribution with mean 0 and variance Σ. |A| denotes the determinant of A. ${∥x∥}_{2}$ denotes the $\ell_{2}$ norm of x. vec[A] denotes the vectorization of the matrix A formed by stacking its columns into a single column vector. $A^{⊤}$ denotes the transpose of A. tr(A) denotes the trace of A.

## 2. Two-Dimensional Reconstruction Methods for HSI Data

As described in the previous section, this section will combine the fast method proposed in our previous work [20] to compress and reconstruct HSI data from two different two-dimensional (2D) slice methods and will provide two 2D reconstruction algorithms for three-dimensional (3D) HSI data. These two methods are the method of expanding 3D data HSI from different bands to 2D data by using a tensor and the method of slicing them into 2D data one by one according to the acquisition mode.

The HSI data experiment was carried out with the repetition of 100 real tests. Each experimental datum was selected from the Salinas Database  (http://www.ehu.eus/ccwintco/index.php/Hyperspectral_Remote_Sensing_Scenes#Salinas-A_scene (accessed on 10 November 2021)) dataset in a 30×50 area, and 20 bands were used, that is, the data matrix was a 30×50×20 3D matrix with N=30,Q=50,M=20. The two 2D reconstruction methods are first introduced in the following sections. The core part of this section shows the direct application of the fast L&S-bSBL method [20]. The same abbreviations as in [20] are used here.

(1) Estimation of x.
  After the Bayesian posterior probability is obtained with the Bayesian rule, the maximum a posteriori (MAP) is used to obtain the estimation of x:
  (1) $$\begin{array}{cc} \hat{x}=\mathrm{vec}\left({\hat{X}}^{⊤}\right)≜\mu_{x} & ={\left(\lambda \Sigma_{0}^{-1}+H^{⊤}H\right)}^{-1}H\Sigma_{0}\\ \; & =\Sigma_{0}H^{⊤}{\left(\lambda I+H\Sigma_{0}H^{⊤}\right)}^{-1}y, \end{array}$$
  where $\Sigma_{0}$ is a block matrix of x, and there are few elements that are non-zero. Moreover, the sparsity of blocks of $\hat{x}$ is determined by $\gamma_{i}\gamma_{j}$. When $\gamma_{k}=0$, the value of the *k*th related block in $\hat{x}$ is zero.
(2) Estimation of λ.
  In order to obtain λ, we simplify the expression of $\Sigma_{0}$ as
  (2) $$\Sigma_{0}=\Gamma ⊗B.$$
  After maximizing the logarithm of the joint probability of x and y, we take the derivative of it with respect to λ:
  (3) $$\lambda \leftarrow \frac{∥y-H\mu_{x}{∥}_{2}^{2}+\lambda^{\left(pre\right)}\left[mn-\mathrm{tr}(\Sigma_{x}\Sigma_{0}^{-1})\right]}{pn}.$$
(3) Estimation of B.
  (4) $$\begin{array}{cc} B\; & =\arg\min_{X}\mathrm{tr}\left[B_{0}^{-1}\left({\mathrm{XX}}^{⊤}+\nabla_{B_{0}^{-1}}\right)\right]+m\log\left|B_{0}\right|\\ \; & =\frac{1}{m}\left(\hat{X}{\hat{X}}^{⊤}+\nabla_{B_{0}^{-1}}\right). \end{array}$$
  where
  (5) $$B_{0}\leftarrow \frac{1}{m}\sum_{i=1}^{m}\frac{\left(\Sigma_{x}^{i}+\mu_{x}^{i}{\left(\mu_{x}^{i}\right)}^{⊤}\right)}{\gamma_{i}^{2}},$$
  (6) $$\nabla_{B^{-1}}=\sum_{i=1}^{m}B-BH_{i}^{⊤}{\left(H\Sigma_{0}H^{⊤}+\lambda I\right)}^{-1}H_{i}B.$$
(4) Estimation of Γ.
  The algorithm calculates the singular values of X through singular value decomposition (SVD) for each slice of the HSI data and sorts the singular values in descending order. Depending on the singular value of the sequence, we can obtain the value of the corresponding $\gamma_{i}$(i=1,⋯, m). The $\gamma_{i}$ corresponding to the larger singular value is 1, or is otherwise 0. For HSI data, a large singular value distribution can be obtained by analyzing only one segment of its 2D slice signal in advance. The expression of Γ is
  (7) $$\Gamma =\left[\begin{array}{cccc} \gamma_{1}\gamma_{1} & \gamma_{1}\gamma_{2} & \cdots & \gamma_{1}\gamma_{m}\\ \gamma_{2}\gamma_{1} & \gamma_{2}\gamma_{2} & \cdots & \gamma_{2}\gamma_{m}\\ \vdots & \vdots & \ddots & \vdots \\ \gamma_{m}\gamma_{1} & \gamma_{m}\gamma_{2} & \cdots & \gamma_{m}\gamma_{m} \end{array}\right].$$
(5) Estimation of $S_{opt}$.

(8) $$X^{opt}\leftarrow x.$$

### 2.1. Two-Dimensional HSI Reconstruction Algorithm—L&S-bSBL (1)

**Figure 1:**
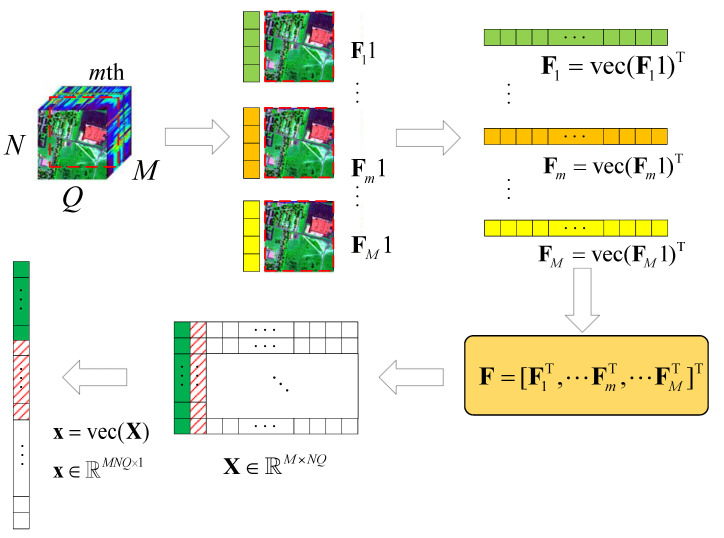
Schematic diagram of the HSI data tensor expanded into 2D data.

Here, we consider a typical HSR scenario (push-broom imaging) in which there are *M* sensors with different wavelengths (called channels or bands; here, we have *M* bands) for acquiring HSI data $F=\left[:,F_{q},:\right]\in R^{MN\times Q},q\in \{1,2,\cdots$, Q} in an N×Q area following the *Q*-dimension, where $F_{q}=[f_{1},\cdots$, $f_{M}{]}^{⊤}\in R^{M\times N\times 1}$. Here, we simply express this as $F_{q}=[f_{1},\cdots$, $f_{M}{]}^{⊤}\in R^{M\times N}$. After all HSI data F are collected, the proposed algorithm performs the following steps on the HSI data, as shown in Figure 1.

(1) According to the different bands *M*, we use the tensor to expand with $F_{1}1,\cdots$, $F_{m}1,\cdots$, $F_{M}1$;
(2) let $F_{m}1,m\in \{1,2,\cdots$, M} turn into $F_{m},m\in \{1,2,\cdots$, M} by using the vec operator;
(3) let $F=[F_{1}^{⊤},F_{2}^{⊤},\cdots$, $F_{M}^{⊤}]$;
(4) obtain the corresponding value of F in the DCT domain $X\in R^{M\times NQ}$.

**Figure 2:**
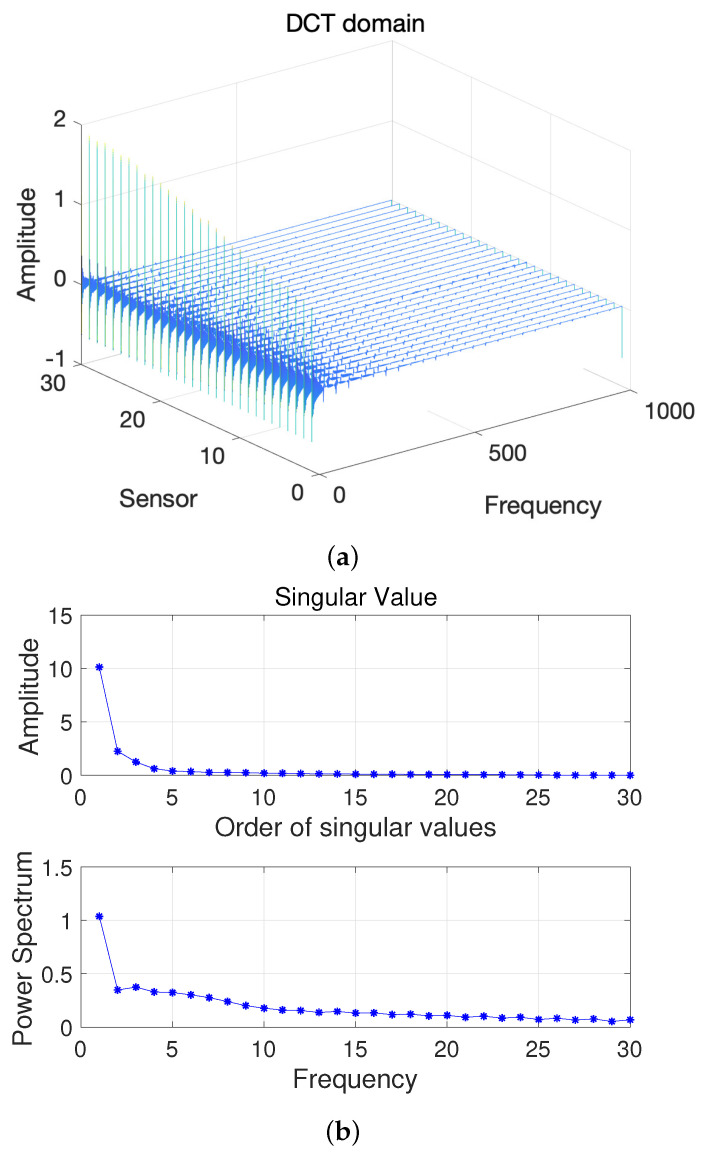
(**a**) Two-dimensional HSI in the DCT domain and (**b**) Singular value vs. Frequency.

**Figure 3:**
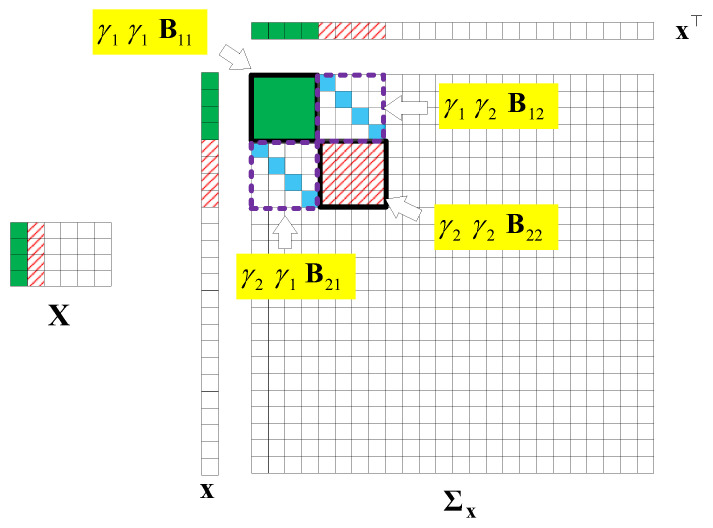
A covariance matrix of 2D HSI data X.

Here, we first conduct a principle component analysis (PCA) on the data F. As shown in Figure 2, we can obtain 2D HSI tensor expansion data F in the sparse domain, which is mainly distributed in the first two columns, as well as the corresponding $\gamma_{1}=1$ and $\gamma_{2}=1$; the other $\gamma_{i},i\in \{2,3,\cdots$, M} is 0. Figure 3 shows a schematic diagram of the covariance matrix of **x**.

For the obtained $X\in R^{M\times NQ}$, the single measurement vector (SMV) model is used to solve the problem of data compression, transmission, and reconstruction. First, the acquired data X are columnarized into x, encoded by the linear mapping matrix H, and then transmitted to the ground receiving station through a wireless channel. Here, we assume that the encoded data will be superimposed with noise e in the process of channel transmission, and the data received at the ground receiving station are denoted as y. Therefore, the mathematical model of the problem can be expressed as y=Hx+e. The proposed algorithm uses the fast L&S-bSBL algorithm in Equations (1), (3), (4), (7) and (8) to iteratively reconstruct the original data x; then, we obtain F, X. Most existing research regards the HSI signal as a low-rank signal to reconstruct, so the typical Bayesian low-rank reconstruction algorithm BARM [21] and the simultaneous low-rank and joint-sparse reconstruction algorithm SS&LR [22] are selected as the algorithms for comparison.

**Figure 4:**
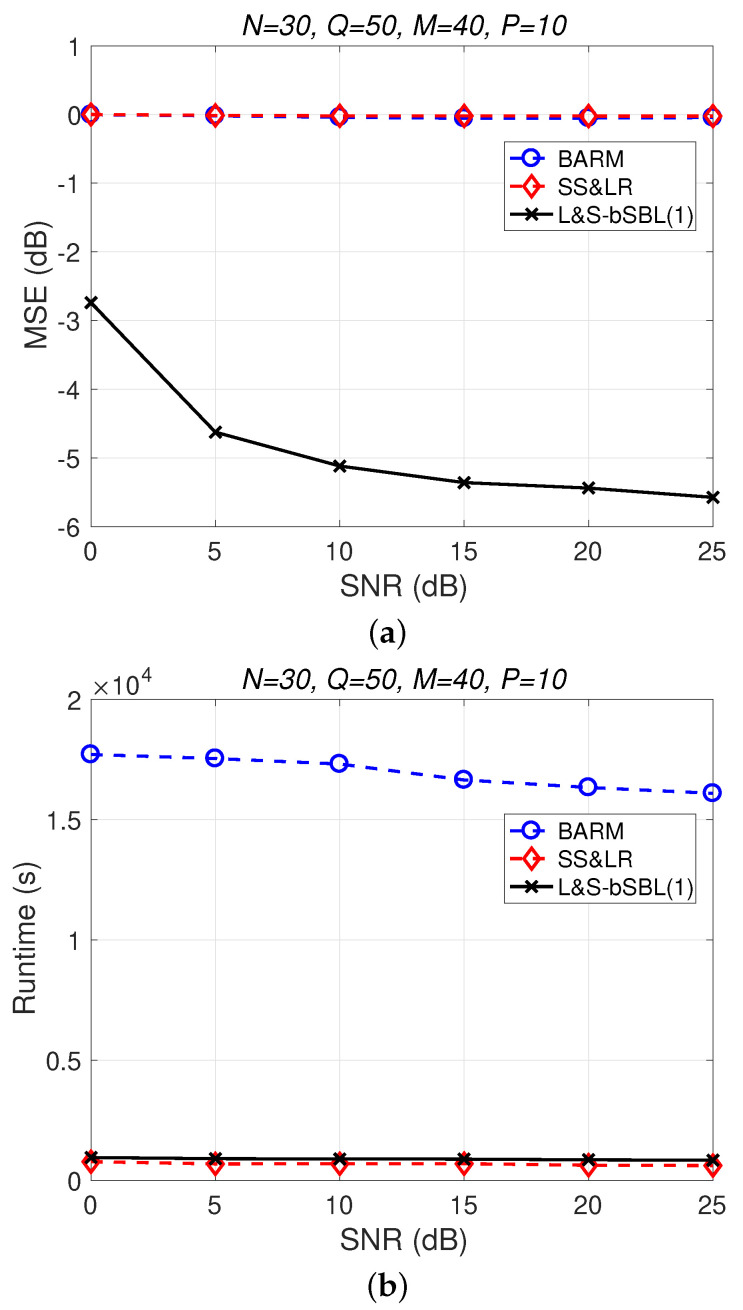
(**a**) MSE vs. SNR and (**b**) runtime vs. SNR for the reconstruction of 2D HSI data.

Figure 4 shows a comparison of the reconstruction performance (including mean squared error (MSE) vs. signal-to-noise ratio (SNR) and runtime vs. SNR) of all algorithms in reconstructing 2D unfolded HSI data. In Figure 4a, we observe that the proposed algorithm L&S-bSBL (1) outperforms all of the other methods because the data expansion method of the proposed algorithm increases the amount of relevant data. As shown in Figure 4b, the proposed algorithm uses less runtime than BARM and almost the same amount as SS&LR. Comprehensive consideration of the two figures shows that the proposed algorithm is better than the other two algorithms in terms of reconstruction performance and computational resource consumption.

### 2.2. Two-Dimensional HSI Reconstruction Algorithm—L&S-bSBL (2)

**Figure 5:**
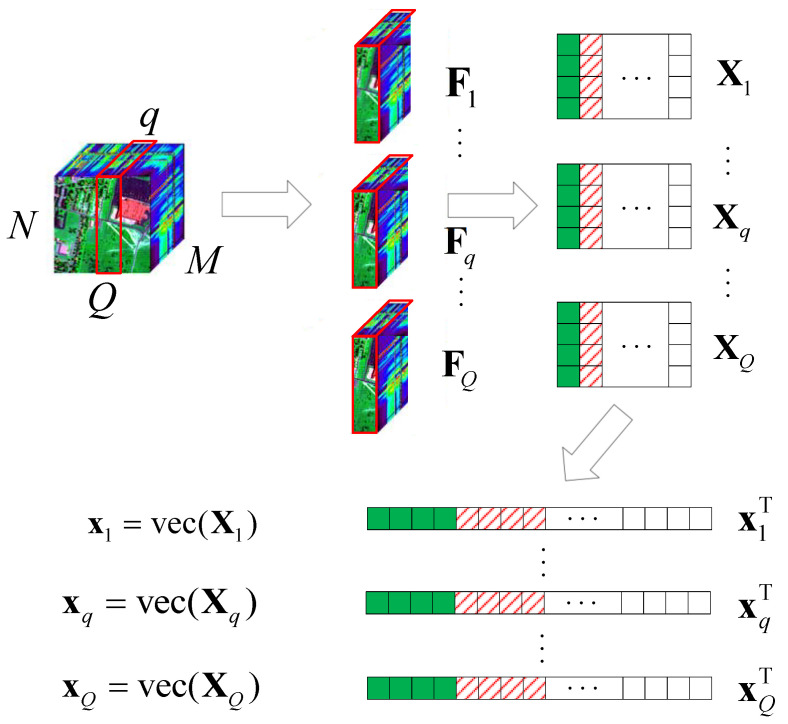
A schematic diagram of the HSI slice decomposition according to the push-broom imaging method following *Q*.

Here, we also consider a typical HSR scenario (push-broom imaging) in which there are *M* sensors with different wavelengths (called channels or bands; here, we have *M* bands) to acquire HSI data $F=\left[:,F_{q},:\right]\in R^{MN\times Q},q\in \{1,2,\cdots$, Q} in an N×Q area following the *Q*-dimension, where $F_{q}=[f_{1},\cdots$, $f_{M}{]}^{⊤}\in R^{M\times N\times 1}$. After all HSI data F are collected, the proposed algorithm performs the following steps on the HSI data, as shown in Figure 5.

(1) Let slices $F_{q}\in R^{M\times N\times 1}$ and q∈{1,2,⋯, Q} as $F_{q}\in R^{M\times N}$;
(2) obtain the corresponding value of $F_{q}\in R^{M\times N}$ in the DCT domain $X_{q}$, q∈{1,2,⋯, Q};
(3) let $X_{q}$ turn into $x_{q}$, q∈{1,2,⋯, Q} by using the vec operator.

**Figure 6:**
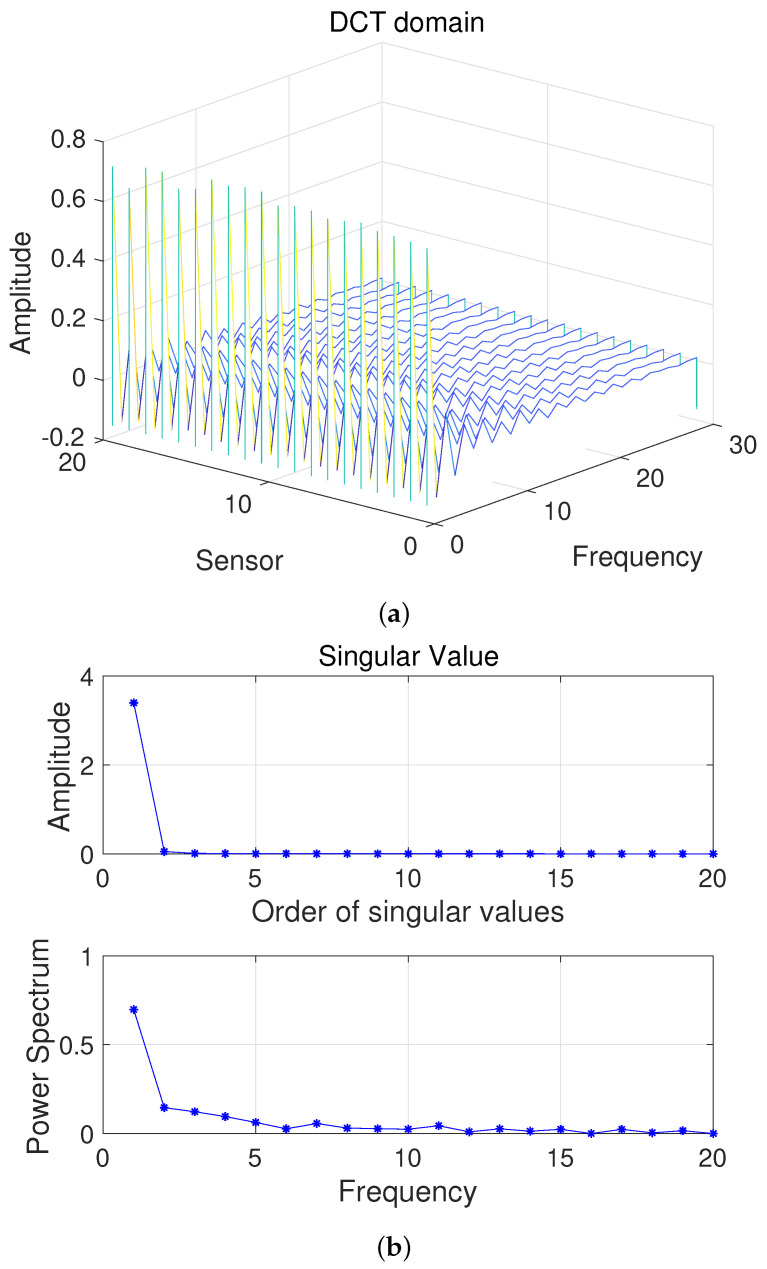
(**a**) Two-dimensional HSI slices in the DCT domain and (**b**) Singular value vs. Frequency.

Here, we first conduct a PCA on the data $F_{q}$. As shown in Figure 6, we can obtain 2D HSI slice data $F_{q}$ in the sparse domain, which is mainly distributed in the first two columns, as well as the corresponding $\gamma_{1}=1$ and $\gamma_{2}=1$; the other $\gamma_{i},i\in \{2,3,\cdots$, M} is 0. The schematic diagram of the covariance matrix of x is similar to that shown in Figure 3.

For the obtained $X_{q}\in R^{M\times N}$, the SMV model is used to solve the problem of data compression, transmission, and reconstruction. Firstly, the acquired data $X_{q}$ are columnarized into $x_{q}$, encoded by the linear mapping matrix H, and then transmitted to the ground receiving station through a wireless channel. Here, we assume that the encoded data will be superimposed with noise $e_{q}$ in the process of channel transmission, and the data received at the ground receiving station are denoted as $y_{q}$. Therefore, the mathematical model of the problem can be expressed as $y_{q}=Hx_{q}+e_{q}$. The proposed algorithm uses the fast L&S-bSBL algorithm in Equations (1), (3), (4), (7) and (8) to iteratively reconstruct the original data x; then, we obtain F, X. Most existing research regards HSI signals as low-rank signals to recover, so BARM and SS&LR are selected as the algorithms for comparison.

**Figure 7:**
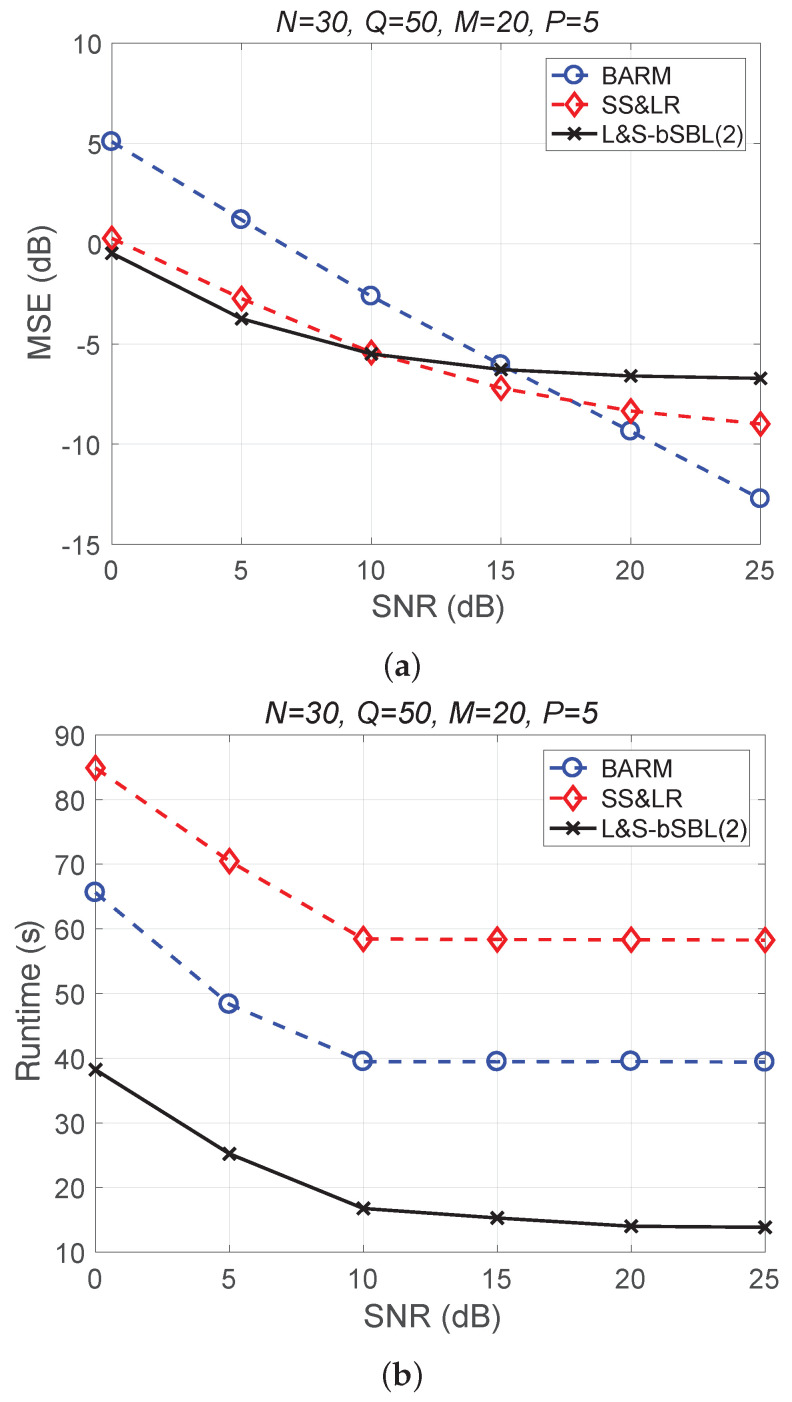
(**a**) MSE vs. SNR and (**b**) runtime vs. SNR for the reconstruction performance of 2D HSI push-brooming slices in all algorithms.

Figure 7 shows a comparison of the reconstruction performance of all algorithms for all *Q* slices. In Figure 7a, we can see that the reconstruction of these slices is acceptable. However, L&S-bSBL (1) has better performance than L&S-bSBL (2) in reconstructing the same data, as shown in Figure 4. The reason is that L&S-bSBL (1) greatly utilizes the correlation between all data and improves the reconstruction performance, while L&S-bSBL (2) only uses the correlation within a certain band, ignoring the correlation between the bands at the same location.

Moreover, L&S-bSBL (2) only takes about 35 s compared with L&S-bSBL (1), which takes 150 s to recover the same data, as shown in Figure 4. This is because L&S-bSBL (1) has a large data dimension for a single operation and L&S-bSBL (2) has a small dimension, and the amount of calculation required for the calculation of the matrix inverse operation is not within an order of magnitude.

## 3. L&S Reconstruction Algorithm Combined with Acquisition Methods

### 3.1. Problem Formulation and Signal Model

**Figure 8:**
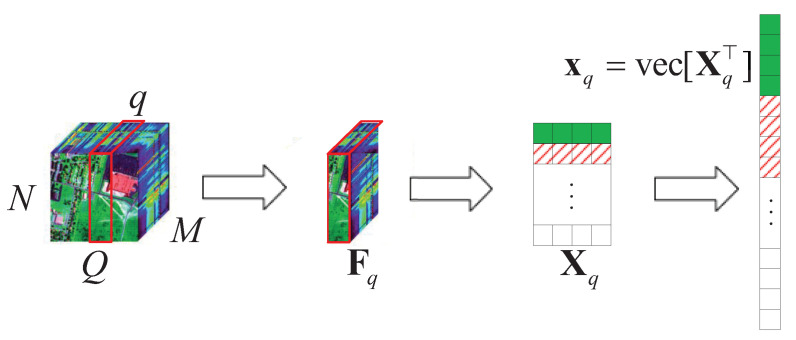
An example of hyperspectral image acquisition.

We consider a typical HSR scenario (push-broom imaging) in which there are *M* sensors with different wavelengths (called channels or bands; here, we have *M* bands) to collect HSI data $F=\left[:,F_{q},:\right]\in R^{MN\times Q},q\in \{1,2,\cdots$, Q} in an N×Q area. Figure 8 shows that a spaceborne spectrometer (push-broom imaging) acquires multi-channel data $F_{q}=[f_{1},\cdots$, $f_{M}{]}^{⊤}\in R^{M\times N}$ with time synchronization among different regions following the *Q*-dimension, where $f_{m}\in R^{N\times 1},m\in \{1,2,\cdots$, M} stands for the data collected by the mth channel sensor and $F_{q}$ is the spatially and temporally correlated data matrix. Then, $F_{q}$ is encoded by linearly mixing with $\Xi_{q}$ and transmitted to a ground receiving station, denoted as $Y_{q}$, after superimposing noise $V_{q}$. Finally, a novel CS method is used to decode all $F_{q}$ at the same time at the ground processing center.

Fortunately, HSI data are highly correlated with the locations and bands of their non-zero elements in a sparse domain. So, we can get an approximately L&S matrix $X_{q}\in R^{M\times N}$ from $F_{q}=\Psi_{q}X_{q}$, where $\Psi_{q}\in R^{M\times M}$ is a sparsifying basis (e.g., DCT matrix or wavelet matrix) [23], and $X=\left[:,X_{q},:\right]\in R^{MN\times Q}$. Thus, we have the following formulation:

(9) $$Y_{q}=\Phi_{q}X_{q}+V_{q},$$

where $\Phi_{q}=\Xi_{q}\Psi_{q}$ is a known dictionary matrix. Here, this problem belongs to the multiple measurement vector (MMV) [24] problem.

Following our recent work [12], we now consider a bSBL framework [25] (i.e., L&S-bSBL) to reconstruct all $X_{q}$. By letting $y_{q}=\mathrm{vec}\left[Y_{q}^{⊤}\right]\in R^{P\times 1}$, $A_{q}=\Phi_{q}⊗I_{n}\in R^{P\times NM}$, $x_{q}=\mathrm{vec}\left[X_{q}^{⊤}\right]\in R^{NM\times 1}$, $v_{q}=\mathrm{vec}\left[V_{q}^{⊤}\right]\in R^{P\times 1}$, after transforming the MMV problem to the block SMV [24] problem, we have

(10) $$y_{q}=A_{q}x_{q}+v_{q}.$$

Then, the original problem becomes

(11) y=Ax+v,

where $y=[y_{1}^{⊤},\cdots$, $y_{Q}^{⊤}{]}^{⊤}\in R^{QP\times 1}$, $A=I_{Q}⊗A_{q}(q\in \{1,2,\cdots$, $Q\})\in R^{QP\times QNM}$, and $I_{Q}\in R^{Q\times Q}$ denotes a Q×Q identity matrix. $x=[x_{1}^{⊤},\cdots$, $x_{Q}^{⊤}{]}^{⊤}\in R^{QNM\times 1}$, $x_{q}$ is the *q*th block in x. The presence of *K* non-zero rows in $X_{q}$ means that there are K×Q non-zero blocks in x. Thus, x is a block sparse vector. $v=[v_{1}^{⊤},\cdots$, $v_{Q}^{⊤}{]}^{⊤}\in R^{QP\times 1}$.

We assume that the noise elements $v_{q}$, q∈{1,⋯, Q} follow an identical and independent distribution with $p\left(v_{q}\right)∼N\left(0,\lambda \right),\forall q$. We define the Gaussian likelihood for problem (10) as

(12) $$p\left(y|x;A,\lambda \right)∼N_{y}|x\left(\mathrm{Ax},\lambda I\right)\propto \exp[-\frac{1}{2\lambda }{∥Ax-y∥}_{2}^{2}],$$

and the prior of x is given by

(13) $$p\left(x;\gamma_{qi},\gamma_{qj},B_{qij},\forall q,i,j\right)∼N_{x}\left(0,\Sigma_{0}\right)\propto \exp\left[-\frac{1}{2}x^{⊤}\Sigma_{0}^{-1}x\right],$$

where $B_{qij}\in R^{N\times N}$ is a covariance matrix between $x_{qi}$ and $x_{qj}$, i,j=1,⋯, *M*. $\Sigma_{0}=I_{Q}$$⊗\Sigma_{q}$, $\Sigma_{q}=\Gamma_{q}⊗B_{qij}$, $\Gamma_{q}=\Gamma_{q0}\Gamma_{q0}^{⊤}$. $\Gamma_{q0}=[\gamma_{q1},\cdots$, $\gamma_{qM}{]}^{⊤}$ is the sparsity pattern vector of $X_{q}$, where the support indicates $\gamma_{qi}\in \left\{0,1\right\}$, i=1,⋯, *M*.

Typically, we can obtain

(14) $$\begin{array}{cc} \; & \Sigma_{0}=\mathrm{diag}\left(\Sigma_{1},\cdots ,\Sigma_{q},\cdots ,\Sigma_{Q}\right),\\ \; & \Sigma_{q}=\left[\begin{array}{cccc} \gamma_{q1}\gamma_{q1}B_{q11} & \gamma_{q1}\gamma_{q2}B_{q12} & \cdots & \gamma_{q1}\gamma_{qM}B_{q1M}\\ \gamma_{q2}\gamma_{q1}B_{q21} & \gamma_{q2}\gamma_{q2}B_{q22} & \cdots & \gamma_{q2}\gamma_{qM}B_{q2M}\\ \vdots & \vdots & \ddots & \vdots \\ \gamma_{qM}\gamma_{q1}B_{qM1} & \gamma_{M}\gamma_{2}B_{qM2} & \cdots & \gamma_{qM}\gamma_{qM}B_{qMM} \end{array}\;\right]. \end{array}$$

**Figure 9:**
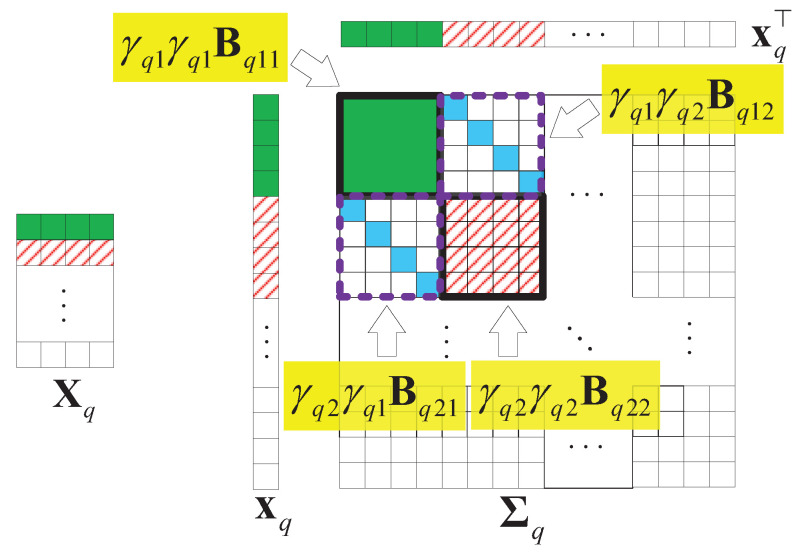
An example of the structure of the covariance matrix $\Sigma_{q}$ of $x_{q}$, $x_{q}=\mathrm{vec}\left[X_{q}^{⊤}\right]$, M=4.

Without losing generality, we assume that $X_{q}$ is a matrix that is low-rank and joint-sparse in columns. Figure 9 illustrates an example of $X_{q}$ and the structure of the covariance matrix $\Sigma_{q}$ of $x_{q}$ with M=4. In the figure, we find that only the first two columns have values, so only $\gamma_{q1}=1$ and $\gamma_{q2}=1$. Thus, the parts related to $\gamma_{q1}$, $\gamma_{q2}$ in $\Sigma_{q}$ are valuable.

### 3.2. Proposed Method

Following our last work [12], we obtain the posterior density of x with the Bayesian rule:

(15) $$p\left(x|y;\lambda ,\gamma_{q}i,\gamma_{q}j,B_{qij},\forall q,i,j\right)∼N_{x}\left(\mu_{x},\Sigma_{x}\right),$$

where the mean $\mu_{x}$ and the covariance $\Sigma_{x}$ can be obtained by

(16) $$\mu_{x}=\frac{1}{\lambda }\Sigma_{x}A^{⊤}y,$$

(17) $$\begin{array}{cc} \Sigma_{x} & ={\left(\Sigma_{0}^{-1}+\frac{1}{\lambda }A^{⊤}A\right)}^{-1}\\ \; & =\Sigma_{0}-\Sigma_{0}A^{⊤}{\left(\lambda I+A\Sigma_{0}A^{⊤}\right)}^{-1}A\Sigma_{0}. \end{array}$$

When all of the hyperparameters $\lambda ,\gamma_{qi},\gamma_{qj},B_{qij},\forall q,i,j$ are given, the maximum a posteriori (MAP) estimate of x can be obtained by

(18) $$\begin{array}{cc} \hat{x}=\mathrm{vec}\left[{\hat{X}}^{⊤}\right]≜\mu_{x} & ={\left(\lambda \Sigma_{0}^{-1}+A^{⊤}A\right)}^{-1}A\Sigma_{0}\\ \; & =\Sigma_{0}A^{⊤}{\left(\lambda I+A\Sigma_{0}A^{⊤}\right)}^{-1}y, \end{array}$$

where $\Sigma_{0}$ is the approximate diagonal matrix obtained by Equation (14), with most block matrices being zeros. It is clear that $\gamma_{qi},\gamma_{qj},\forall q,i,j$ control the sparsity of the blocks of $\hat{x}$. When $\gamma_{qk}=0$, the associated qkth block in $\hat{x}$ becomes zero. In fact, $\gamma_{1i}=\cdots =\gamma_{qi}=\cdots =\gamma_{Qi}$.

Following the bSBL framework [25], to avoid overfitting, we use a common positive definite matrix Σ to model all of the covariance matrices $\Sigma_{q}$. In $\Sigma_{q}$, we also use a common positive definite matrix B instead of $B_{qij}$ and we use a group of $\gamma_{i}\in \left\{0,1\right\}$ instead of all $\gamma_{qi}\in \left\{0,1\right\}$, Γ and instead of $\Gamma_{q}$. From the previous analysis, we can find that the covariance matrices induce the spatiotemporal correlation in the prior density. Thus, Equation (14) can be written as

(19) $$\Sigma_{0}=I_{Q}⊗\Gamma ⊗B.$$

Using the Bayesian strategy, we maximize the marginal probability p(x,y) of x:

(20) $$\max_{B\in N^{+},\Gamma \geq 0}\int p(y|x;A,\lambda )p(x;\Gamma ,B)dx,$$

which is equivalent to minimizing the cost function of −2logp(y;λ,Γ,B):

(21) $$L\left(\Gamma ,B,\lambda \right)=y^{⊤}\Sigma_{y}^{-1}y+\log|\Sigma_{y}|,$$

where $N^{+}$ denotes a set of N×N positive definite matrices.

(22) $$\Sigma_{y}=A\Sigma_{0}A^{⊤}+\lambda I,\;\Sigma_{0}=I_{Q}⊗\Gamma ⊗B.$$

Here, $\Sigma_{y}$ denotes the covariance of y given Γ and B.

Simply, Θ≡{Γ,B,λ}; thus, (21) turns into

(23) $$L\left(\Theta \right)=y^{⊤}\Sigma_{y}^{-1}y+\log|\Sigma_{y}|.$$

Firstly, x is treated as a hidden variable in the expectation maximization (EM) formulation that proceeds, and we maximize

(24) $$\begin{array}{cc} Q(\Theta )= & E_{x|y;\Theta^{\left(pre\right)}}\left[\log\left(p(y|x;\lambda )p(x;\Gamma ,B)\right)\right]\\ = & E_{x|y;\Theta^{\left(pre\right)}}\left[\log p\left(y|x;\lambda \right)\right]\\ \; & +E_{x|y;\Theta^{\left(pre\right)}}\left[\log p\left(x;\Gamma ,B\right)\right], \end{array}$$

where $\Theta^{\left(pre\right)}$ denotes the hyperparameters that are estimated in the previous iteration.

To estimate λ—only the first term in the Q function is correlated with λ—it can be simplified as

(25) $$\begin{array}{cc} Q(\lambda )=\; & E_{y|x;\Theta^{\left(pre\right)}}\left[\log p(y|x;\lambda )\right]-\frac{QP}{2}\log\lambda \\ \; & -\frac{1}{2\lambda }\left[∥y-A\mu_{x}{∥}_{2}^{2}+\lambda^{\left(pre\right)}\left[QNM-\mathrm{tr}\left(\Sigma_{x}\Sigma_{0}^{-1}\right)\right]\right], \end{array}$$

where $\lambda^{\left(pre\right)}$ denotes the estimation of λ in the previous iteration. When we calculate the derivative of Equation (25) over λ and set it equal to zero, λ is obtained:

(26) $$\lambda \leftarrow \frac{∥y-A\mu_{x}{∥}_{2}^{2}+\lambda^{\left(pre\right)}\left[QNM-\mathrm{tr}(\Sigma_{x}\Sigma_{0}^{-1})\right]}{PN}.$$

To estimate Γ and B, $\Gamma =\mathrm{diag}(\gamma_{1}^{2},\cdots ,\gamma_{M}^{2})$ is first assumed, where diag(·) denotes a diagonal matrix operator. Notice that only the second term in Equation (24) is related to Γ and B. So, we can simplify the Q function (24) to

(27) $$Q\left(\Gamma ,B\right)=E_{x|y;\Theta^{\left(pre\right)}}\left[\log p\left(x;\Gamma ,B\right)\right],$$

Then, we have

(28) $$\begin{array}{cc} Q(\Gamma ,B)\propto & -\frac{QN}{2}\log\left(|\Gamma |\right)-\frac{QM}{2}\log\left(|B|\right)\\ \; & -\frac{1}{2}\mathrm{tr}\left[\left(\Gamma^{-1}⊗B^{-1}\right)\left(\Sigma_{x}+\mu_{x}\mu_{x}^{⊤}\right)\right]. \end{array}$$

**Figure 10:**
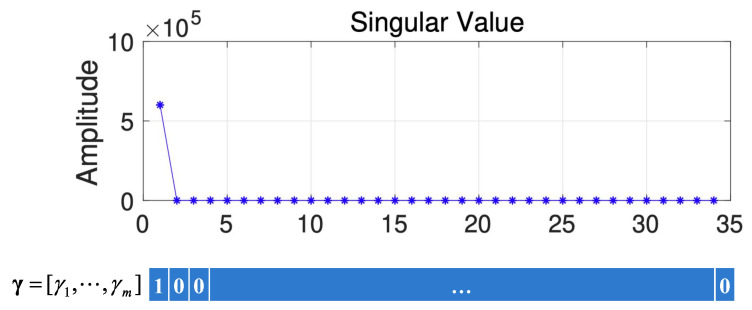
The schematic diagram of how $\gamma_{i}$ is obtained.

To obtain the values of $\gamma_{i}$, we use the feature given in [20]. Specifically, the values of $\gamma_{i}$ corresponding to the larger singular values (the larger singular values are defined as singular values that are larger than 10% of the largest singular value) are 1; otherwise, they are 0. Figure 10 gives a schematic diagram of how $\gamma_{i}$ is obtained from HSI data. From Figure 10, we can find that only the first singular value is larger, so $\gamma_{1}=1$.

To estimate B, $\mu_{x}$ and $\Sigma_{x}$ are plugged into Equation (28). So, we can obtain the gradients of Equation (28) over B, and then we can obtain $B^{\left(pre\right)}$.

(29) $$B^{\left(pre\right)}\leftarrow \frac{1}{M}\sum_{i=1}^{M}\frac{\left(\Sigma_{x}^{i}+\mu_{x}^{i}{\left(\mu_{x}^{i}\right)}^{⊤}\right)}{\gamma_{i}^{2}}.$$

Thus, we will get $\Gamma^{\left(pre\right)}$. Using the same method, we can get $\lambda^{\left(pre\right)}$. Finally, we get $\Theta^{\left(pre\right)}$. Here, $A^{\left(pre\right)}$ denotes an initial value of A.

In order to get a closed form of Θ, we employ standard upper bounds to solve Equation (21), which is known as a non-convex optimization problem leading to an EM-like algorithm. For the first and second terms of L(Γ,B), we compute their respective bounds.

For the first term in Equation (21), we can obtain

(30) $$y^{⊤}\Sigma_{y}^{-1}y\leq \frac{1}{\lambda }{∥y-\mathrm{Ax}∥}_{2}^{2}+x^{⊤}\Sigma_{0}^{-1}x,$$

where equality is obtained when x satisfies Equation (18).

For the second term, we can obtain

(31) $$\begin{array}{cc} \log|\Sigma_{y}| & \equiv QM\log|B|+\log|\lambda A^{⊤}A+\Sigma_{0}^{-1}|\\ \; & \leq QM\log|B|+\mathrm{tr}[B^{-1}\nabla_{B^{-1}}]+C, \end{array}$$

where, for the second term $\log|\lambda A^{⊤}A+\Sigma_{0}^{-1}|$, a first-order approximation is used to approximate it with a bias term *C*. The equality will hold when the gradient satisfies

(32) $$\nabla_{B^{-1}}=\sum_{m=1}^{M}B-BA_{m}^{⊤}{\left(A\Sigma_{0}A^{⊤}+\lambda I\right)}^{-1}A_{m}B,$$

where $A=[A_{1},\cdots ,A_{M}]$ and $A_{m}\in R^{QP\times QN}$. Finally, by using the upper bounds of Equations (30) and (31) and $\nabla_{B^{-1}}$, we can obtain the optimal B in a closed form:

(33) $$B^{opt}=\arg\min_{x}x^{⊤}\Sigma_{0}^{-1}x+\mathrm{tr}\left[B^{-1}\nabla_{B^{-1}}\right]+QM\log\left|B\right|.$$

Starting with $B=B^{\left(pre\right)}$, we iteratively compute Equations (18), (32) and (33), then obtain an estimation for B and a corresponding estimation for x given by Equation (18). Here, the proposed method is outlined in Algorithm 1.
**Algorithm 1** Proposed method**Source data analysis**calculate singular values of $X_{q}$ by using SVD;obtain $\gamma_{i}$, i=1,⋯,M by using singular values;**Input**y,A;  **Output**X;**Initialize**    **assume**  $\Gamma =\mathrm{diag}(\gamma_{1}^{2},\cdots ,\gamma_{M}^{2})$;$iters=0,\;\delta =10^{-6}$;maxiterationnumber=500;Set λ,B by $\lambda =10^{-10}$, B=ones(N,N);compute λ,B from Equations (26) and (33); $\Sigma_{0}\leftarrow I_{Q}⊗\Gamma ⊗B$;**While**$∥X-\hat{X}{∥}_{2}^{2}\geq \delta$compute $\hat{X}$ by Equation (18); compute $\nabla_{B^{-1}}$ by Equation (32);compute $B^{opt}$ by Equation (33); iters=iters+1;**if**  iters≥500       **STOP**;**end if****EndWhile**Get the best $B^{opt}$ and X.

### 3.3. Simulation Experiments

In this section, we present the results with HSI data in order to compare the performance of the proposed method with that of the prior state-of-the-art LRMR [5] and SS&LR [22] methods. For HSI data, 100 continuous-time trials were run. In each trial, data of a 30×50 two-dimensional area and 20 bands from the Salinas Database (available at http://www.ehu.eus/ccwintco/index.php/Hyperspectral_Remote_Sensing_Scenes#Salinas-A_scene (accessed on 10 November 2021)) and Indian Pines (available at http://www.ehu.eus/ccwintco/index.php/Hyperspectral_Remote_Sensing_Scenes#Indian_Pines (accessed on 10 November 2021)) were used. Thus, the data matrix was a 30×50×20 matrix.

**Figure 11:**
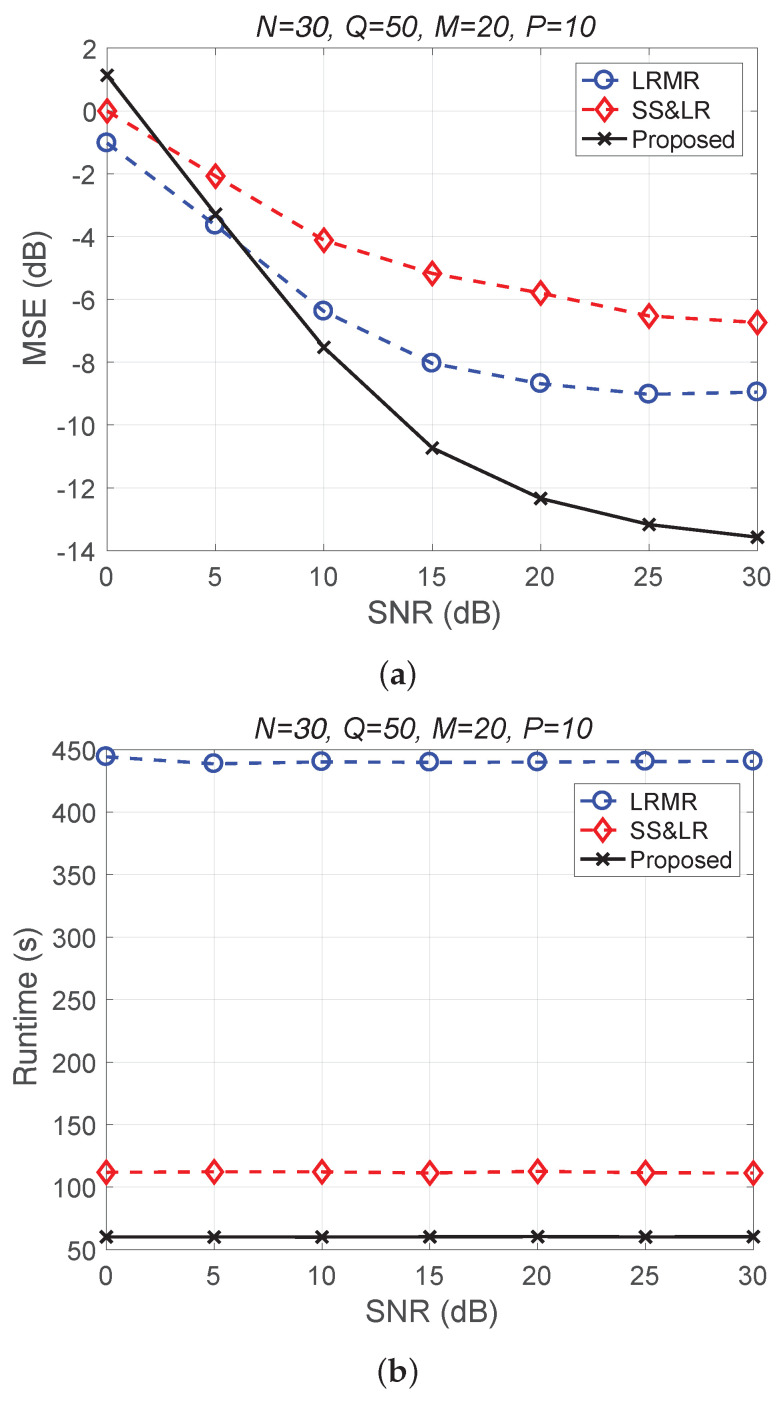
(**a**) MSE vs. SNR and (**b**) runtime vs. SNR for the reconstruction of the Salinas data.

**Figure 12:**
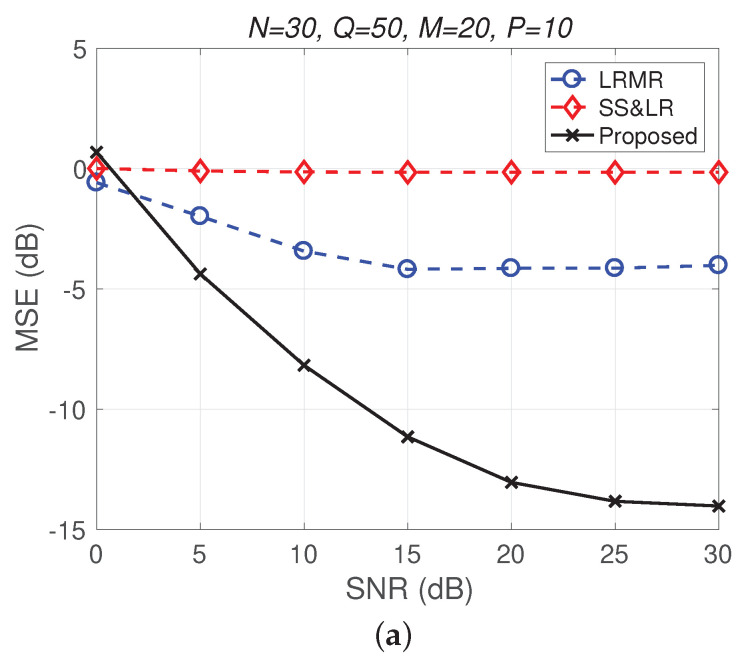

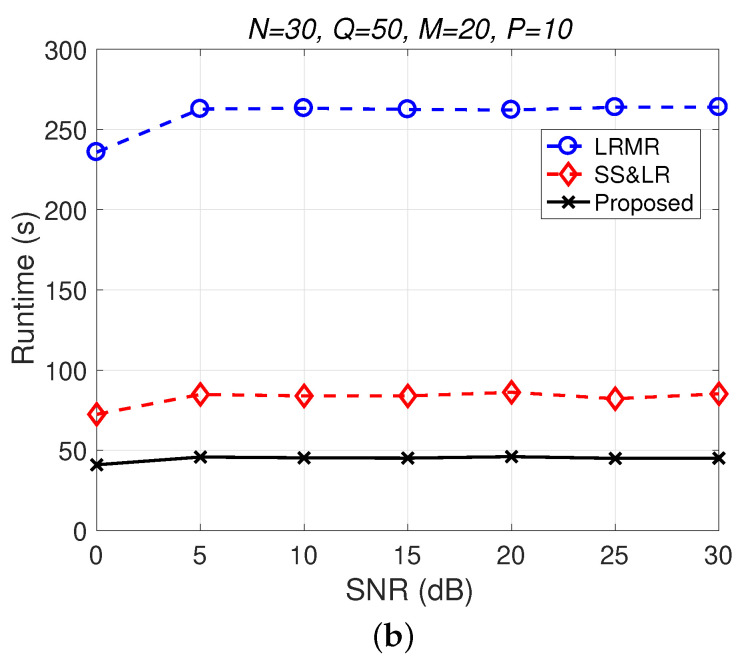
(**a**) MSE vs. SNR and (**b**) runtime vs. SNR for the reconstruction of the Indian Pines data.

Figure 11 and Figure 12 plot the performance in terms of MSE versus SNR and runtime versus SNR, respectively. Here, P=10, so the ratio of compression is P/NM=1/60. In Figure 11a, we can observe that proposed method outperforms all of the other methods in terms of MSE; e.g., at SNR=20 dB, we note that the proposed method achieves reconstruction gain at least 5 dB greater than those of the other methods. Figure 11b shows that the proposed method uses less runtime than the others. In Figure 12, we can observe that the effect of SS&LR is not good, but our proposed method works well and outperforms all of the other methods in terms of MSE; e.g., at SNR=15 dB, we note that the proposed method achieves a reconstruction gain at least 6 dB greater than those of the other methods. The proposed method also uses less runtime than the others.

**Figure 13:**
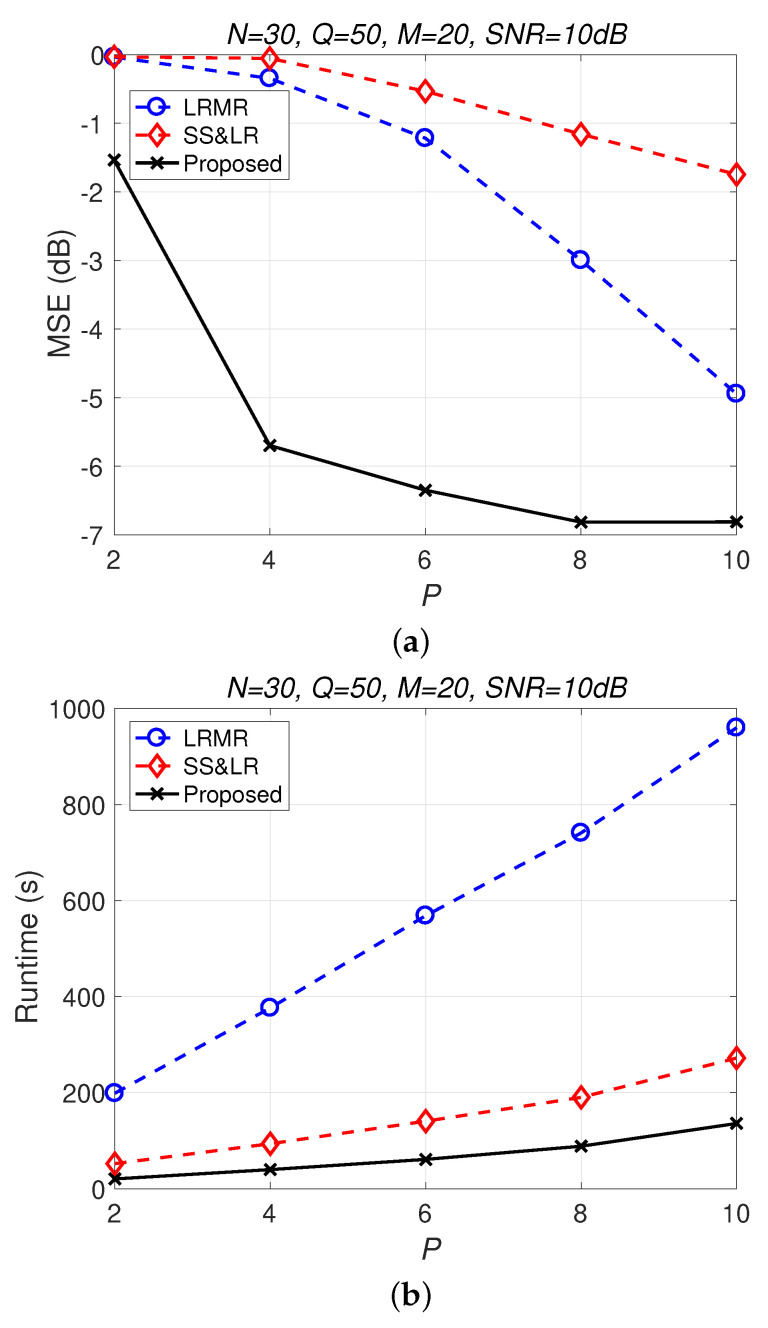
(**a**) MSE vs. *P* and (**b**) runtime vs. *P* with different compression ratios for the reconstruction of the Salinas data.

**Figure 14:**
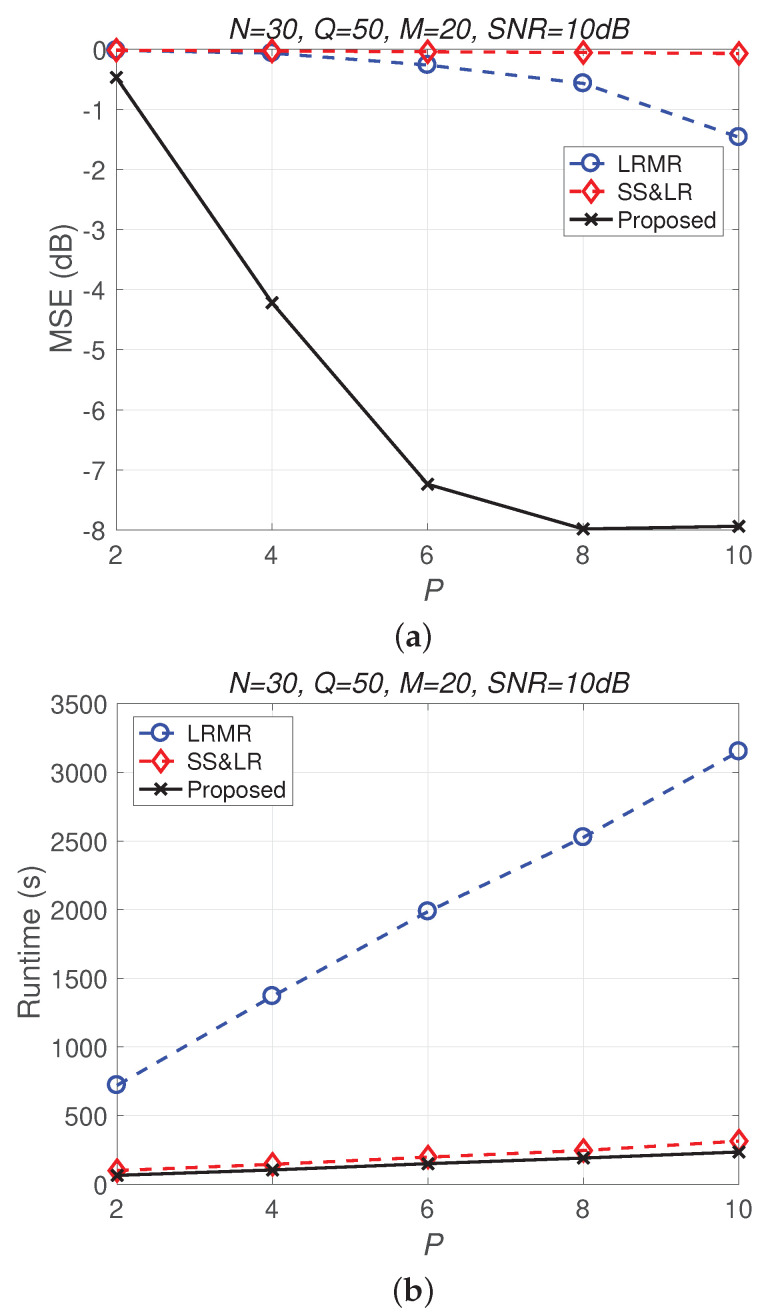
(**a**) MSE vs. *P* and (**b**) runtime vs. *P* with different compression ratios for the reconstruction of the Indian Pines data.

Figure 13 and Figure 14 compare all of the methods for different values of compression *P*. Figure 13a shows that most of the MSE curves fall as *P* increases. As the compression ratio P/NM decreases (from 1/300 to 1/60), the reconstruction error becomes smaller, affecting the signal reconstruction performance. Fortunately, the proposed algorithm yields better performance in all cases and has a higher compression ratio with the same reconstruction performance. Figure 13b shows that the proposed method consumes less computation time at the same compression ratio. From Figure 14, we observe that the effects of SS&LR and LRMR are not good, but our proposed method outperforms all of the other methods in terms of MSE; e.g., at P=6, we note that the proposed method achieves a reconstruction gain at least 7 dB greater than those of the other methods. The proposed method also uses less runtime than the others.

**Figure 15:**
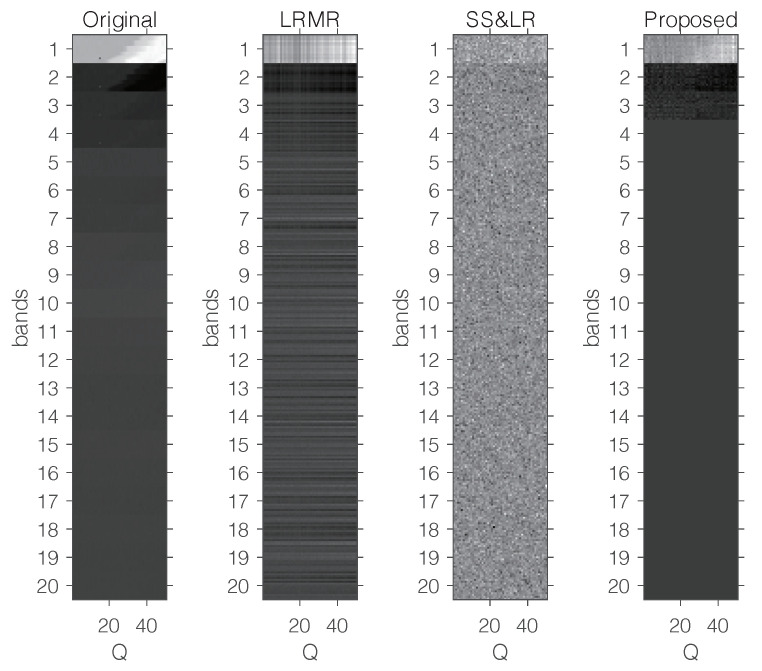
Visual comparison with a fixed compression ratio P/NM=1/30 and 20 bands.

Figure 15 shows some visual reconstruction results obtained with the three methods. As expected, the proposed method preserves more fine details and better visual results than the others.

**Figure 16:**
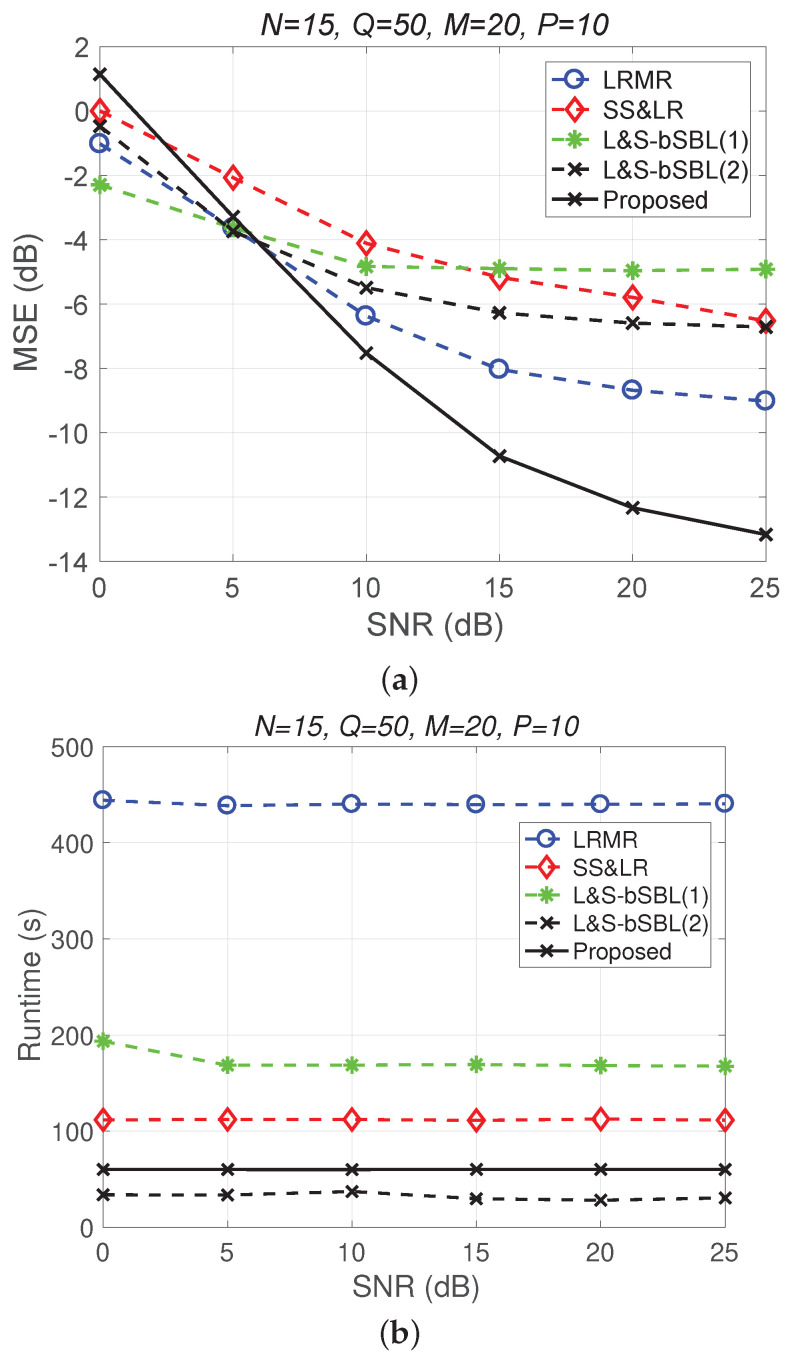
(**a**) MSE vs. SNR and (**b**) runtime vs. SNR for only the performance of each algorithm from the perspective of signal recovery, regardless of the HSI data acquisition method.

Figure 16 only shows a performance comparison chart of the various algorithms from the perspective of data recovery, and it does not start from the perspective of data collection methods, as in the beginning of this section. It can be seen in Figure 16 that the proposed 3D joint reconstruction algorithm has better performance and less time consumption than the other algorithms. What needs to be explained here is that the three methods—the proposed 3D HSI method, L&S-bSBL (1) method, and L&S-bSBL (2) method—are, in terms of the compression ratio: P/(NM)=1/60, P/(NQ)=1/150, and P/N=1/3.

## 4. Discussion

From Section 3.3, the simulation results demonstrate that the proposed method is superior to LRMR and SS&LR methods in terms of reconstruction accuracy and computational burden under the same SNR and compression ratio. This is because LRMR only focuses on the reconstruction of low-rank signals, while SS&LR focuses on the reconstruction of low-rank and sparse signals, but it only uses the spatial relationship or interband relationship of HSI signals and does not combine the signal acquisition process.

Based on our present work, some issues that could be addressed in future research can be summarized as follows. One open problem is that of reducing, as much as possible, the computational complexity of the proposed method. In particular, unmanned aerial vehicles (UAVs) are used to monitor fields, such as in farmland monitoring, environmental monitoring, etc. While our approach has significant reconstruction performance, there is still a long way to go before we can apply it to future portable hardware. We can also take advantage of the properties of HSI data with higher-dimensional structures to design an effective Bayesian learning-based reconstruction method; namely, tensor models could be considered to compress and reconstruct HSI data. Furthermore, we may also consider the design of a sensor selection method for HSI acquisition that reduces the number of sensors in different locations that are needed so as to extend the life and energy savings of sensors.

## 5. Conclusions

In this work, based on the fast method proposed in our previous work, two methods for 2D compression and reconstruction of 3D HSI data are given. Then, starting from the HSI acquisition method, we analyzed the push-brooming acquisition of data slices in the hyperspectral image acquisition process, and by assuming that the HSI data met the L&S model, a joint CS compression and reconstruction method for spatial and spectral correlation was studied and an L&S structure was proposed. The proposed method combines data reconstruction with HSI data acquisition. The simulation results show that the proposed method has better performance than the other two existing methods and the two proposed 2D reconstruction methods.

## Data Availability

Not applicable.
